# PcWRKY11, an II-d WRKY Transcription Factor from *Polygonum cuspidatum*, Enhances Salt Tolerance in Transgenic *Arabidopsis thaliana*

**DOI:** 10.3390/ijms23084357

**Published:** 2022-04-14

**Authors:** Guowei Wang, Xiaowei Wang, Hongping Ma, Haili Fan, Fan Lin, Jianhui Chen, Tuanyao Chai, Hong Wang

**Affiliations:** 1 College of Life Sciences, University of Chinese Academy of Sciences, Yuquan Road, Beijing 100049, China; wangguowei19@mails.ucas.ac.cn (G.W.); wangxiaowei15@mails.ucas.ac.cn (X.W.); mahongping20@mails.ucas.ac.cn (H.M.); fanhaili18@mails.ucas.ac.cn (H.F.); linfan20@mails.ucas.ac.cn (F.L.); chengjianhui21@mails.ucas.ac.cn (J.C.); 2 Institute of Genetics and Developmental Biology, Chinese Academy of Sciences, Beichen West Road, Beijing 100101, China

**Keywords:** *Polygonum cuspidatum*, PcWRKY11, salt stress, ROS, transgenic *Arabidopsis thaliana*

## Abstract

Being an invasive plant, *Polygonum cuspidatum* is highly resilient and can survive in unfavorable environments for long periods; however, its molecular mechanisms associated with such environmental resistance are largely unknown. In this study, a WRKY transcription factor (TF) gene, *PcWRKY11*, was identified from *P. cuspidatum* by analyzing methyl jasmonate (MeJA)-treated transcriptome data. It showed a high degree of homology with WRKY11 from *Arabidopsis thaliana*, containing a WRKY domain and a zinc finger structure and II-d WRKY characteristic domains of HARF, a calmodulin-binding domain (C-motif), and a putative nuclear localization signal (NLS) through sequence alignment and functional element mining. qPCR analysis showed that the expression of PcWRKY11 can be induced by NaCl, osmotic stress, and UV-C. In this study, we also found that overexpression of *PcWRKY11* in *A. thaliana* could significantly increase salt tolerance. To explore its possible molecular mechanism, further investigations showed that compared with the wild type (WT), under salt stress, the transgenic plants showed a lower malondialdehyde (MDA) content, higher expression of *ascorbate peroxidase (APX*) and *superoxide dismutase* (*SOD*), and higher enzyme activity of peroxidase *(*POD), superoxide dismutase (SOD), and catalase (CAT). Moreover, the transgenic plants also showed higher expression of Δ^1^-pyrroline-5-carboxylate synthase (*AtP5CS*), and higher contents of proline and soluble sugar. Taken together, these results indicate that *PcWRKY11* may have a positive role in plants’ adaptation to salinity conditions by reducing reactive oxygen species (ROS) levels and increasing osmosis substance synthesis.

## 1. Introduction

Soil salinization is a serious unfavorable environmental factor that hinders plant seed germination, growth, and fruiting. Currently, more than 800 million hectares of land are affected by soil salinization worldwide, and the ratio is still increasing [1]. Excessive soil salinity causes plants to be subjected to osmotic and ionic toxicity stress, leading to growth inhibition, development changes, and severely hindered metabolism. Osmotic and ion stress can also cause secondary stress to plants, including the accumulation of toxic compounds and the destruction of nutrient balance. ROS, such as hydroxyl radicals, hydrogen peroxide, and superoxide anions, accumulate in plant cells, which can severely damage cell structures and macromolecules, such as enzymes, DNA, and lipids, which in turn affect plant growth and even cause death [2,3].

To adapt to such challenges, plant respond to salt stress through gene expression, transcriptional regulation, and signal transduction [4]. Among them, transcriptional regulation is the most important regulation part of plants’ tolerance against salt stress. TFs are integral in linking salt-sensory pathways to many tolerance responses. Core sets of TF family genes are differentially expressed in response to elevated external salinity [5], including the WRKY [6], MYB [7], basic helix-loop-helix (bHLH) [8], basic leucine zipper (bZIP) [9], and NAC [10] families. These TFs in turn regulate the expression levels of various genes that may ultimately affect the level of salt tolerance in plants [11].

The WRKY TF family is one of the largest TF families in higher plants. WRKY is named after the conserved WRKYGQK sequence, which contains about 60 amino acid residues at the N-terminus. The members of this family contain one or two WRKY domains at the N-terminus and a zinc finger motif at the C-terminus and are divided into three categories, namely groups I, II, and III. The first one contains two WRKY domains and a C_2_H_2_ zinc finger motif, the second one includes a WRKY domain and a C_2_H_2_ zinc finger motif, and the third one contains a WRKY domain and a C_2_HC zinc finger motif [12]. Phylogenetic tree analysis of AtWRKY based on their WRKY domains showed that group II can be further divided into five distinct subgroups (II a–e). The II-d subgroup possesses two domains in common in addition to the WRKY domain and the zinc finger motif: the so-called HARF domain of unknown function, and the C domain, which was shown to mediate calcium-dependent interaction with calmodulin. Calcium is an important second messenger in plant signal transduction pathways, including the response to abiotic stress. This suggests that the II-d group WRKY may also be involved in plant response to abiotic stresses [13].

In recent years, increasingly more research reports have pointed out that WRKY plays an important role in a variety of stress response processes in plants, especially in responses to salt stresses [14,15]. For example, land cotton GhWRKY17 and *Arachis hypogaea* AhWRKY75 improved salt tolerance in transgenic plants by upregulating several genes related to the ROS scavenging system [16,17]. Likewise, *Ipomoea batatas IbWRKY2* overexpression improved transgenic *A. thaliana* proline contents and SOD activity after salt treatments [18]. Wheat TaWRKY13 enhanced salt tolerance in transgenic *Arabidopsis* and after salt treatment, *TaWRKY13* overexpressing *A. thaliana* lines had a longer root and a larger root surface area than the control [19].

*P. cuspidatum* is regarded as an invasive plant in Europe and North America because of its aggressive growth, allelopathy, and strong abiotic stress tolerance. *P. cuspidatum* could also cause huge ecological (biodiversity loss) and economic problems (infrastructure damage) [20,21]. Studies have shown that soil salinity plays an important role in promoting or limiting the spread of alien species [22,23,24,25,26]. *P. cuspidatum* exhibits tolerance to high salt treatment, which is the essential factor of being an invasive plant [27]. Therefore, it is necessary to explore the salt tolerance mechanism of *P. cuspidatum*. However, currently, there are only a few studies on the stress resistance mechanisms of *P. cuspidatum*. For example, Bao et al. (2018) reported that PcWRKY33 negatively regulates the salt tolerance of transgenic plants by downregulating the induction of stress-related genes and by increasing the level of cellular ROS [28]. Recently, a new member of the AP2/ERF TF gene, *PcDREB2A*, was identified through transcriptome data analysis, and it enhanced drought tolerance in transgenic plants [29].

Studies have shown that salt tolerance is critical for *P. cuspidatum* to be a successful invasive plant [27]. To further elucidate this salt resistance mechanism, PcWRKY11 was first identified in *P. cuspidatum* by analyzing MeJA-treated transcriptome data, then overexpressed in *A. thaliana* via *Agrobacterium*-mediated transformation. Our results demonstrated that PcWRKY11 plays a positive role in plant adaptation to salinity conditions by reducing ROS levels and increasing osmosis substance synthesis.

## 2. Results

### 2.1. Identification and Sequence Analysis of PcWRKY11

The transcriptome database of *P. cuspidatum* treated with MeJA (accession number: PRJNA626400) was constructed in our laboratory. Through association analysis, we obtained four splicing sequences annotated as MYB/WRKY related to metabolism and stress, and further studied the function of one of them in this study. According to the known sequence, specific primers were designed, and gene fragments were amplified with *P. cuspidatum* cDNA as a template. The full length of the cDNA was 1014 bp, encoding a WRKY domain protein of 338 amino acids.

**Figure 1:**
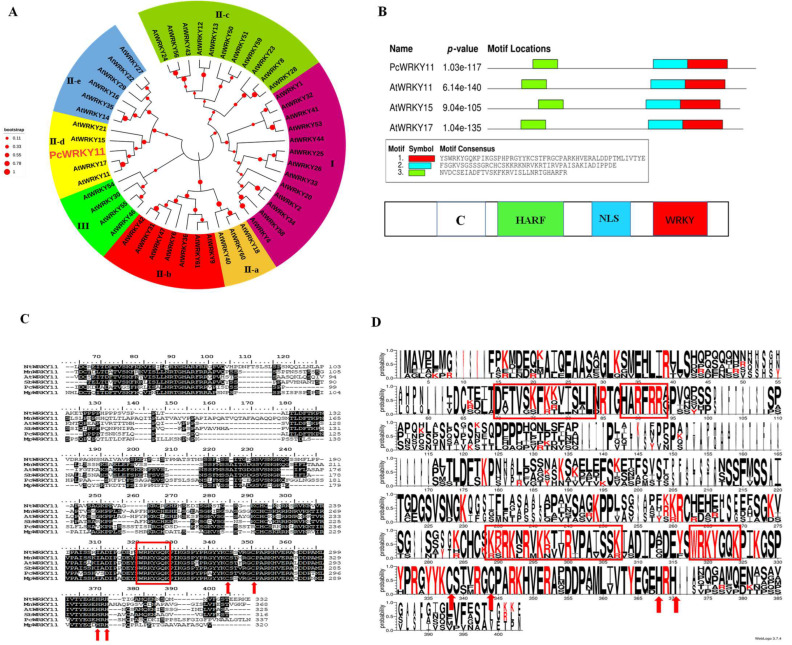
Bioinformatics analysis of *PcWRKY11*. (**A**) Phylogenetic tree of the PcWRKY11 and AtWRKY protein family. The amino acid sequences were subjected to Clustal W using the neighbor-joining method in MEGA X; PcWRKY11 is marked with red enlarged font; At, *Arabidopsis thaliana*; Pc, *Polygonum cuspidatum*. (**B**) The schematic diagram of the II-d WRKY conserved domain. White: binding domain with calmodulin, green: II-d WRKY characteristic domain, blue: nuclear localization domain, red: WRKY characteristic domain. (**C**) Sequence alignment of PcWRKY11 to other plant WRKY11. Identical amino acids are shaded in color, the WRKYGQK conservative sequence is outlined by a box, and the C and H residues in the zinc-finger motif are marked by an arrow; Nt, *Nicotiana tabacum*; Mn, *Morus notabilis*; At, *Arabidopsis thaliana*; Sb, *Soy beans*; Pc, *Polygonum cuspidatum;* Mp, *Mucuna pruriens.* (**D**) Visualization of the result of the sequence similarity of PcWRKY11 to the other plant WRKY11. The larger the letter, the more conservative the amino acid. Shared conserved domains are boxed in red.

The phylogenetic tree analysis of the protein sequence with the *A. thaliana* WRKY protein family showed that it presents a 42% similarity with AtWRKY11 of *A. thaliana* (A0A1P8B8D0), and was preliminarily classified into the II-d subfamily of the WRKY family (Figure 1A). Due to the high degree of homology with AtWRKY11, the new WRKY gene was preliminarily named *PcWRKY11*. To further mine useful information from the PcWRKY11 protein sequence, functional element mining was performed by comparing the PcWRKY11 protein sequence with typical ⅠⅠ-d WRKYs, AtWRKY11, AtWRKY15, and AtWRKY17 using the MEME combinatorial tool. The analysis result showed that the PcWRKY11 protein sequence contains a typical nuclear localization signal sequence (KRKRK) preceded by the WRKY domain. This protein is predicted to have a possible role in the nucleus as a TF (Figure 1B).

To further confirm the nomenclature correctness of *PcWRKY11*, sequence comparison with WRKY11 from other species, including NtWRKY11 (A0A1U7W174), AtWRKY11 (A0A1P8B8D0), SbWRKY11 (A0A445EXZ3), MpWRKY11 (A0A371FYH7), and MnWRKY11 (A0A1U7W174), was performed. The results showed that the gene contains a WRKY signature domain (marked by a red box) and a C_2_H_2_-type zinc finger structure (marked by red arrows), which conforms to the ⅠⅠ WRKY family characteristics (Figure 1C). Meanwhile, by visually analyzing the homology alignment results in Figure 1C, we found that the PcWRKY11 protein sequence contained a conserved C functional domain (DTVFISL) in the 65 to 100 amino acid interval, which served as the ⅠⅠ-d WRKY subfamily characteristic domain, associated with calmodulin binding (Figure 1D). In summary, *PcWRKY11* was correctly named and submitted to GenBank (MZ734625).

### 2.2. Expression Analysis of PcWRKY11

**Figure 2:**
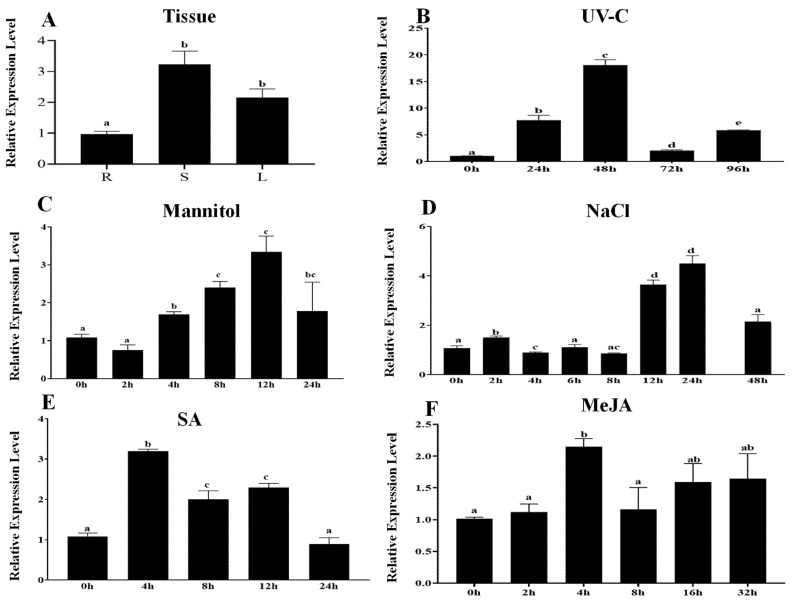
Expression level of *PcWRKY11* in *P. cuspidatum.* (**A**) Tissue expression. R, root; S, stem; L, leaf. (**B**) UV-C. (**C**) Mannitol. (**D**) NaCl. (**E**) SA. (**F**) MeJA. The plants without any treatment were used as the control, and *PcNDUFA13* was the reference gene. Each column stands for a point of time after treatment, and each experiment was repeated at least twice. (**B**–**F**): The leaves of 1-month-old seedlings were used in gene expression analysis. Any two bars within the graph with a common letter mean they are not significantly different (*p* > 0.05).

To further investigate the expression pattern of *PcWRKY11*, qRT-PCR was conducted to detect the transcription levels of *PcWRKY11* in different tissues and in response to various abiotic stresses. The results of the expression levels in different tissues show that the expression of *PcWRKY11* is relatively high in stems and leaves, with the highest expression found in stems, which was 3-fold more than the expression in roots (Figure 2A).

Among the stress conditions, five stress treatments of MeJA, ultraviolet C (UV-C), high salt (NaCl), salicylic acid (SA), and mannitol were selected to analyze the expression pattern of *PcWRKY11*. Under different stress conditions, the expression of *PcWRKY11* induced by UV-C was the most significant, and it was increased by about 18-fold within 0–48 h, reaching a peak at 48 h (Figure 2B). Under the MeJA treatment, the expression level of *PcWRKY11* increased by about 2-fold at 4 h after the treatment and did not change too much in the subsequent 4–32 h (Figure 2F). Under the treatment of 300 mM mannitol, the expression of *PcWRKY11* gradually increased within 2–12 h but declined within 12–24 h (Figure 2C). Under the treatment of 300 mM NaCl, the expression level of *PcWRKY11* gradually increased within 2–24 h and reached a peak at 24 h at about a 4-fold increase but began to decline after 24 h (Figure 2D). Under the SA treatment, the expression of *PcWRKY11* increased about 3-fold after 4 h of treatment, and then began to fall within 4–24 h (Figure 2E). In summary, under hormone treatment, the expression of *PcWRKY11* reached a peak within the short time period of 4 h. Under stress treatment, the expression of this gene often required 24 h or longer to peak. The response of *PcWRKY11* to the UV-C, NaCl, and mannitol treatment was more noticeable.

### 2.3. Characterization of PcWRKY11 as A Transcription Factor

**Figure 3:**
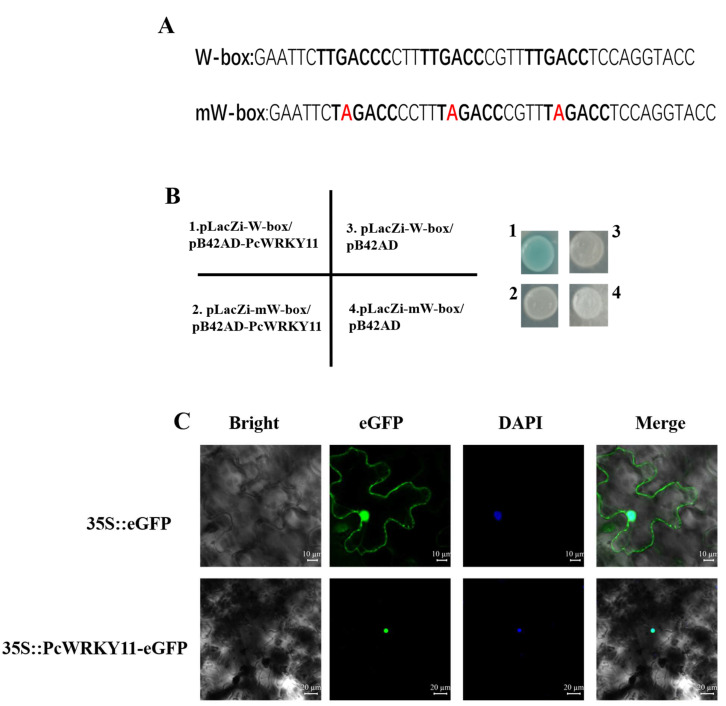
Analysis of *PcWRKY11* transcriptional activation activity. (**A**) Nucleotide sequence of W-box and mW-box. Red letters represent mutant nucleotides (**B**) Yeast one-hybrid assay. The chromogenic results of the yeast one-hybrid assay. 1, *pLacZi-W-box*/*pB42AD-PcWRKY11*; 2, *pLacZi-mW-box*/*pB42AD-PcWRKY11*; 3, *pLacZi-W-box*/*pB42AD*; 4, *pLacZi-mW-box*/*pB42AD*. (**C**) Subcellular localization of PcWRKY11 in tobacco cells. PcWRKY11-eGFP fusion protein and eGFP control were transiently expressed in *N. benthamiana* epidermal cells. A confocal laser scanning microscope was used for observation.

Based on reports that WRKY TFs modulate gene expression by interacting with the cis-element W-box to regulate transcription [30], the yeast one-hybrid assay was performed to verify this characterization of PcWRKY11 protein. Two-nucleotide strands with a three-tandem repeated W-box sequence (TTGACC) and three repeated mutations on the W-box sequences (TAGACC) were synthesized, respectively (Figure 3A). Then, the two nucleotide chains were used to construct the yeast expression vectors *pLacZi-W-box* and *pLacZi-mW-box*, and used to co-transform EGY48 yeast cells with the yeast expression vector *pB42AD-PcWRKY11*. The defective medium SD/-Trp/-Ura was used to screen and obtain the positive strains. Then, the positive strains were scraped with a toothpick and smeared on the X-gal-containing defective medium SD/-Trp/-Ura. The results showed that only the yeast co-transformed with *pLacZi-W-box* and *pB42AD-PcWRKY11* turned blue while the yeast co-transformed with *pLacZi-mW-box* and *pB42AD-PcWRKY11* did not turn blue (Figure 3B). These results showed that PcWRKY11 can bind to W-box to activate the expression of the downstream *LacZ* gene, and this binding is specific.

Bioinformatics analysis of the PcWRKY11 protein sequence predicted its potential location in the nucleus, similar to most other TFs. To verify the prediction, the constructed *pBI121-PcWRKY11-eGFP* and the control *pBI121-eGFP* vectors were transformed into *Agrobacterium tumefaciens* GV3101, respectively, using the freeze-thaw method. The transformed *A. tumefaciens* with the target gene and the control were separately injected into *Nicotiana* *benthamiana* leaves. Under the fluorescence confocal microscope, the green fluorescent protein (GFP) was used as a control and distributed throughout the cell while the fluorescence of the PcWRKY11-GFP fusion protein was only distributed in the nucleus similar to the nucleus after 4,6-diamidino-2-phenylindole (DAPI) staining (Figure 3C). These findings confirmed that PcWRKY11 is located in the nucleus.

### 2.4. PcWRKY11 Enhances Salt Tolerance of Transgenic Arabidopsis

To further investigate the function of *PcWRKY11* in plants, *PcWRKY11* was overexpressed in *A. thaliana* under the control of the *CaMV35S* promoter via *Agrobacterium*-mediated transformation. Transgenic lines were obtained by kanamycin (Kan) selection (50 mg/L) and further confirmed by PCR (data not shown). Then, semi-quantitative RT-PCR analysis was performed to detect the Kan resistance transgenic plants and WT (Supplementary Figure S1). Based on the result of RT-PCR analysis, 3 lines, namely OE1, OE4, OE8, were selected for the subsequent experiments, and these 3 lines were cultured to the T3 generation to obtain homozygous transgenic *A. thaliana* for the following experiments.

qPCR analysis showed that the expression of *PcWRKY11* was significantly induced by salt stress (Figure 2D). In addition, PcWRKY11 belongs to the ⅠⅠ-d subfamily WRKY, which contains the conserved C motif. This motif binds to calmodulin in a Ca^2+^-dependent manner to regulate various biological processes, including the response to salt stress. Therefore, the salt tolerance of PcWRKY11 transgenic *A. thaliana* was investigated.

**Figure 4:**
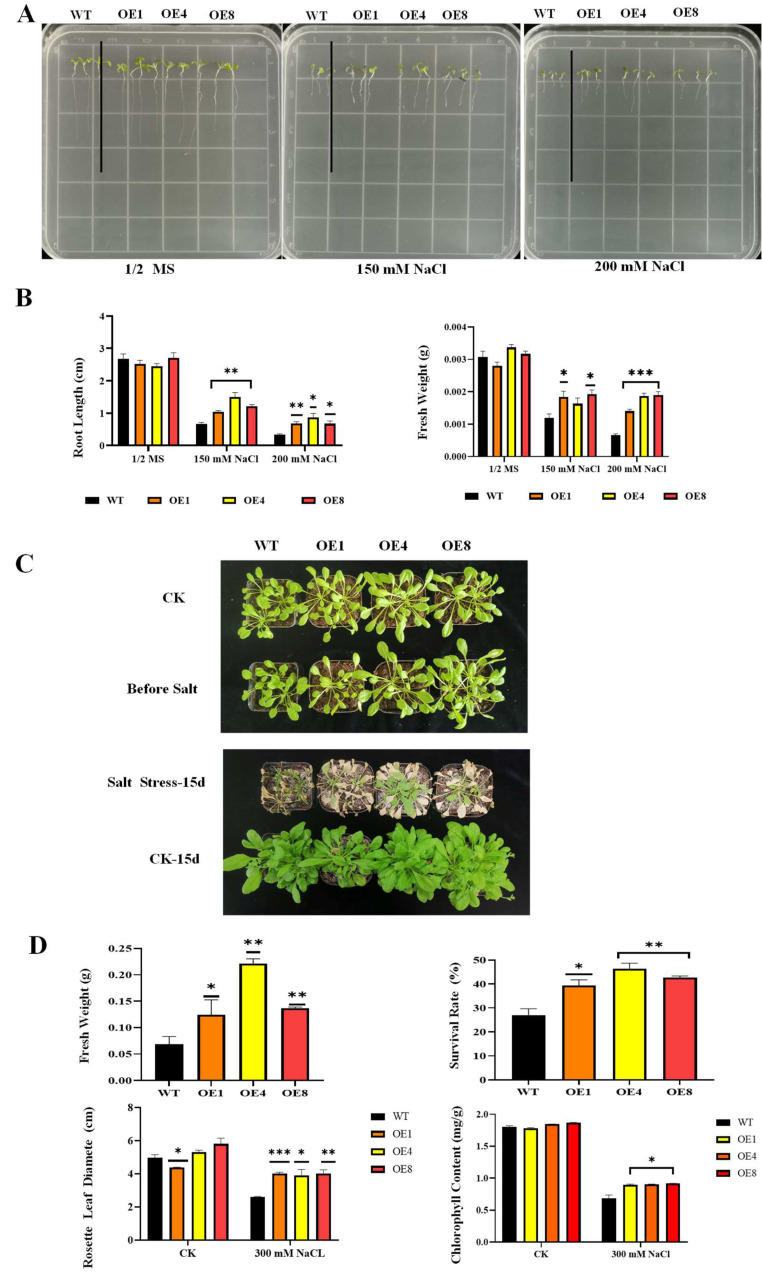
Overexpression of *PcWRKY11* in *A. thaliana* improved salt tolerance. (**A**) Transgenic and wild-type plants grown on 1/2× MS medium with different NaCl concentrations. (**B**) Graphical representation of the root length and fresh weight in (**A**). (**C**) Phenotype of 4-week-old transgenic and wild-type plants after salt treatment (300 mM NaCl, 15 days). (**D**) Statistical analysis of the rosette leaf diameter, fresh weight, survival rate, and chlorophyll content in (**C**). The mean values and standard errors were calculated with three independent experiments. *, **, and *** represent significance level of *p* < 0.05, *p* < 0.01, and *p* < 0.001, respectively; WT, wild-type plants; OE, overexpressed plants; CK, under normal conditions.

We conducted root length experiments on 1/2 MS solid medium containing different concentrations of NaCl (0, 150, and 200 mM). The results of the root length experiment showed that both WT and transgenic *A. thaliana* remained approximately the same on 1/2 MS medium without NaCl. On medium containing 150 mM NaCl, the root growth of the WT was severely inhibited compared with the transgenic lines, and this inhibition was even greater on the medium containing 200 mM NaCl (Figure 4A). The statistical results of the fresh weight showed that the fresh weight of WT was significantly reduced compared with the transgenic lines, and this difference was more significant as the concentration of NaCl increased (Figure 4B).

To further investigate the function of *PcWRKY11* in plant vegetative growth, 4-week-old seedlings of WT and transgenic *A. thaliana* were watered with 300 mM NaCl for 15 days. Before NaCl treatment, the growth of *PcWRKY11* transgenic lines and WT showed no significant difference. After NaCl treatment for 15 days, the growth of both *PcWRKY11* transgenic lines and WT was significantly inhibited, but the WT plants were more severely inhibited than the transgenic lines (Figure 4C).

To further confirm the improvement of *PcWRKY11* overexpression on plant growth, the fresh weight, survival rate, rosette leaf diameter, and chlorophyll content of 4-week-old *PcWRKY11* transgenic lines and WT after salt stress were analyzed. The results showed that the fresh weight, rosette leaf diameter, chlorophyll content, and survival rate of transgenic lines under salt stress were all higher than that of WT plants (Figure 4D). These results indicated that *PcWRKY11* overexpression improved plant growth, as indicated by the rosette leaf growth and chlorophyll synthesis, of transgenic lines, and improved survival compared to WT under salt stress. In summary, the experimental results show that overexpression of *PcWRKY11* increased the salt resistance of transgenic plants.

### 2.5. Analysis of Oxidative Damage in PcWRKY11 Overexpressed Plants

**Figure 5:**
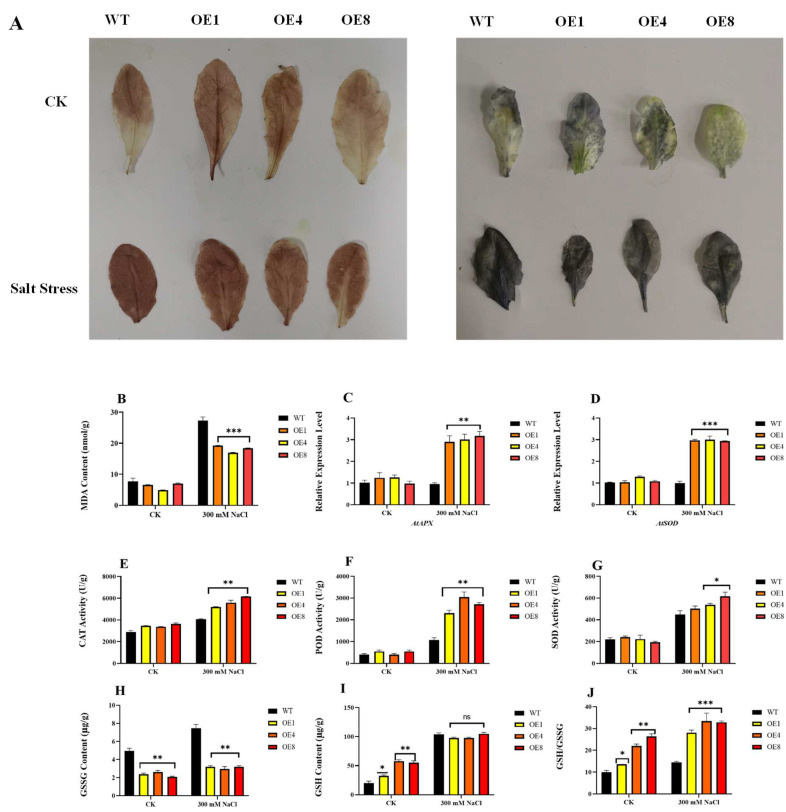
Oxidative damage indexes after overexpressing *PcWRKY11* *A. thaliana* under salt stress. (**A**) Staining under salt stress in overexpressed *PcWRKY11* plants. Leaves of transgenic and wild-type plants after DAB staining for H_2_O_2_ detection under normal conditions and salt treatment. Leaves of transgenic and wild-type plants after NBT staining for O_2_^−^ detection under normal conditions and salt treatment. (**B**) MDA content of transgenic and wild-type plants under normal conditions and salt treatment. (**C**) Relative expression analysis of *AtAPX.* (**D**) Relative expression analysis of *AtSOD.* (**E**) The enzyme activities of CAT. (**F**) The enzyme activities of POD. (**G**) The enzyme activities of SOD. (**H**) GSSG content of transgenic and wild-type plants under normal conditions and salt treatment. (**I**) GSH content of transgenic and wild-type plants under normal conditions and salt treatment. (**J**) GSH to GSSG ratio of transgenic and wild-type plants under normal conditions and salt treatment. The mean values and standard errors were calculated with three independent experiments. *, **, and *** represent a significance level of *p* < 0.05, *p* < 0.01, and *p* < 0.001 respectively; ns, *p* > 0.05, no significant difference; WT, wild-type plants; OE, overexpressed plants CK, under normal conditions.

Salt stress can cause accumulation of ROS in plants, mainly H_2_O_2_ and O^2−^. To further investigate the role of *PcWRKY11* in response to salt stress, we detected the levels of H_2_O_2_ and O^2−^ by 3,3′-diaminobenzidine (DAB) and nitroblue tetrazolium (NBT) staining in WT and transgenic lines after salt treatment. The results of the DAB and NBT staining on detached leaves showed that the leaves of each plant in the control group under normal growth conditions had no obvious staining while the DAB and NBT staining of the transgenic lines after salt treatment was lighter in color than WT, indicating that WT accumulated more ROS and suffered greater damage than *PcWRKY11* transgenic *A. thaliana* under the same salt stress conditions (Figure 5A). Quantitative analysis of the staining results is shown in Supplementary Figure S2.

Excessive ROS accumulation can cause oxidative damage to plant cells, and the concentration of MDA is an important physiological indicator of the degree of oxidative damage to cell membranes. The MDA concentration was tested, and the results showed that MDA was significantly increased in both the WT and transgenic lines after salt treatment, but the concentration increase was significantly higher in WT than in the transgenic lines (Figure 5B). To further clarify the mechanism of salt resistance in the transgenic lines, the relative expression of antioxidant enzyme genes was examined in the transgenic and WT lines after salt stress. The results showed that the expression of *AtAPX* and *AtSOD* in the transgenic lines increased by an average of 3-fold and 2-fold compared to those in the WT, respectively (Figure 5C,D). We also examined the enzyme activities of CAT, SOD, and POD, and the results show that the activities of CAT, SOD, and POD in the transgenic lines were higher than those in the WT after the same salt treatment (Figure 5E–G).

In addition, the glutathione (GSH) and oxidized glutathione (GSSG) contents of the transgenic lines and WT were examined after salt treatment. Figure 5H–J shows that the transgenic lines had a higher GSH/GSSG ratio, which indicates that the transgenic lines contain more reducing substances and can cope with salt-induced oxidative stress better. These results indicate that the transgenic lines improved salt tolerance by enhancing antioxidant enzyme-like activities and antioxidant synthesis under salt stress.

### 2.6. Analysis of Osmotic Stress in PcWRKY11 Overexpressed Plants

Salt stress can also cause osmotic stress to plants. Under osmotic stress, plants synthesize some osmotic-regulating substances, such as proline, betaine, soluble sugar, soluble protein, etc. Among them, the content of proline is one of the most important indicators affecting the ability of plants to tolerate salt stress.

**Figure 6:**
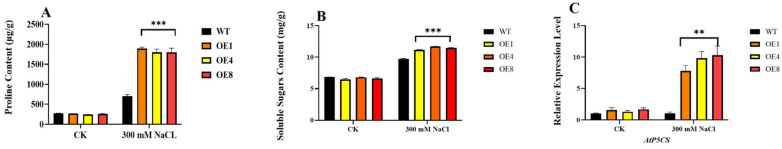
Osmotic stress analysis of *PcWRKY11* overexpression plants under salt stress. (**A**) Proline content in the wild-type and transgenic lines under normal conditions and the salt treatment condition. (**B**) Soluble sugar content in the wild-type and transgenic lines under normal conditions and salt treatment conditions. (**C**) The expression of the proline synthesis gene *AtP5CS* in plants. The mean values and standard errors were calculated with three independent experiments. **, and *** represent a significance level of, *p* < 0.01, and *p* < 0.001, respectively; WT, wild-type plants; OE, overexpressed plants CK, under normal conditions.

To further elucidate the mechanism of *PcWRKY11* improvement of plant salt resistance, we detected the content of proline in transgenic plants and WT plants under the 300 mM NaCl treatment. The results show that significantly more proline was produced by transgenic lines than WT lines under the same salt stress (Figure 6A). In addition, we also detected the content of soluble sugar in transgenic and WT plants at the same time. The results show that significantly more soluble sugar was produced by transgenic lines than WT lines under high salt stress (Figure 6B). To further clarify the differences between transgenic lines and WT under osmotic stress, qPCR was used to detect the gene expression of *AtP5CS*, which is the limiting enzyme gene in the proline synthesis process. The results showed that under high salt stress, the expression of *AtP5CS* in the transgenic lines increased about 8–11-fold, compared with WT (Figure 6C). These results suggest that *PcWRKY11* may improve the salt tolerance of transgenic plants by increasing osmolytes in vivo.

## 3. Discussion

*P. cuspidatum* is listed among the 100 worst invasive alien species in the world [27]. It shows strong adaptability and tolerance to a wide range of stresses, such as high temperature, salt, cold, drought, waterlogging, burning, heavy metals, various soil types, etc. [31,32]. However, few molecular biological studies on the identification of TFs and the possible mechanism of stress resistance in *P. cuspidatum* have been conducted.

MeJA is an endogenous messenger molecule that plays an important role in regulating responses to biotic and abiotic stresses in plants [33,34]. In order to investigate the stress resistance mechanism of *P. cuspidatum*, we established the transcriptome data of *P. cuspidatum* treated with MeJA and obtained a WRKY family TF, whose expression was significantly increased after MeJA treatment. According to its similarity with AtWRKY11, it was named PcWRKY11. The results of multiple sequence alignment showed that this gene contains a WRKY signature domain and a C_2_H_2_ type zinc finger structure, which conforms to the ⅠⅠ group WRKY family characteristics (Figure 1). The PcWRKY11 protein sequence also contains a conserved C functional domain of 65 to 100 amino acids, which serves as the ⅠⅠ-d WRKY subfamily signature domain and is implicated in calmodulin binding [13]. Ca^2+^ is an important second messenger for plants in response to external stresses, suggesting that PcWRKY11 may be involved in plants’ response to external stresses [35]. The subcellular localization assay and yeast one-hybrid analysis showed that PcWRKY11 was mainly localized in the nucleus and could bind to W-box to activate the expression of the downstream gene (Figure 3). These results suggest that PcWRKY11 is a new WRKY in *P. cuspidatum*.

PcWRKY11 has highly similar protein sequences with AtWRKY11 (42%), and previous studies showed that the expression of *AtWRKY11* was significantly increased after the 100 mM NaCl treatment [36]. In addition, *AtWRKY11* and *AtWRKY17* knockout mutants (single mutant and double mutant) resulted in slower germination and root growth under salt stress compared with WT, and double mutants were more severe than single mutants. These results indicated that AtWRKY11 and AtWRKY17 were involved in plant salt stress resistance [37]. To further explore the possible functional network of this gene, we analyzed the AtWRKY11 sequence using the STRING protein database (Supplementary Figure S3. The results showed that AtWRKY11 could interact with the peroxidase superfamily protein AT1G14550, which belongs to the POD family, which is functionally annotated to remove H_2_O_2_ in response to environmental stress, such as pathogen attack and oxidative stress. Among WRKY11 of other species, *OsWRKY11* transgenic rice was reported to have stronger resistance to drought and two serious diseases, thus increasing yields in stress environments [38]. *Medicago sativa MsWRKY11* overexpressed in soybean was reported to increase the salt resistance of transgenic plants by increasing the contents of chlorophyll, proline, soluble sugar, SOD, and CAT, and reducing the contents of MDA, H_2_O_2_, and O^2−^ [39].

The sequence similarity between PcWRKY11 and other plants WRKY11 suggests that this gene may play a role in plant salt tolerance. To investigate this issue, *PcWRKY11* was overexpressed in the model plant *Arabidopsis* under the control of the CaMV35S promoter via *Agrobacterium*-mediated transformation. Analysis of phenotypic and physiological parameters showed that the transgenic lines had a higher root length, survival rate, fresh weight, diameter of rosette leaves, and chlorophyll content under salt stress than WT (Figure 4E). These results indicate that *PcWRKY11* overexpression increased the salt tolerance of transgenic plants.

An increasing number of studies have shown that WRKYs improve plant salt tolerance by removing ROS to reduce salt-induced oxidative damage. For instance, *Salix linearistipularis SlWRKY28* overexpressed in *Populus davidiana × P. bolleana* significantly upregulated salt resistance by increasing the APX activity of transgenic plants [40]. Overexpression of *Zea mays ZmWRKY104* improved salt stress tolerance by alleviating salt-induced ROS accumulation and MDA content in transgenic plants [41]. Consistent with previous studies, the ROS levels of the *PcWRKY11* transgenic lines in this study were lower than those of WT as indicated by DAB and NBT staining (Figure 6A). Generally, ROS contents in plants are maintained in a dynamic balance. However, stress disrupts this homeostasis, and causes lipid membranes to decrease or lose their original functions due to peroxidation and deacylation, which will in turn damage the membrane system and metabolic processes of plants and cause damage and destruction of biological macromolecules, such as proteins and nucleic acids, leading to cell death [42]. To decouple the injurious effects of ROS species on plant cells, plants have evolved a series of antioxidant systems to scavenge oxygen damage, which mainly includes SOD, CAT, POD, and so on. In this study, the qPCR results show that the expression of oxidative stress-related genes (*AtAPX*, *AtPOD*) in the transgenic lines was significantly higher than that in the WT lines (Figure 5C,D). In addition, the antioxidant enzyme activities of CAT, SOD, and POD of the transgenic lines were higher than those of WT (Figure 5E–G). Furthermore, the transgenic lines had a higher GSH/GSSG ratio, indicating that intracellular ROS were degraded in the transgenic lines under high salt stress (Figure 5H–J). These results suggest that *PcWRKY11* can improve the salt tolerance of transgenic lines by enhancing the ROS scavenging ability.

Previous studies have also shown that plants can improve stress tolerance by accumulating osmotic substances, such as soluble sugar and free proline, to regulate the cell osmotic potential and protect the cell structure [43]. Our results indicated that both the free proline and soluble sugar contents of *PcWRKY11* transgenic lines were higher than those of the WT plants under salt stress (Figure 6A,B), and qPCR revealed that the transgenic lines had higher *AtP5CS* expression than the WT lines (Figure 6C). This is consistent with previous studies showing that the accumulation of proline and soluble sugars could increase cellular osmolality, thereby improving plant salt tolerance [44].

In conclusion, a novel II-d WRKY TF, *PcWRKY11*, was identified from *P. cuspidatum* in this study. Overexpression of *PcWRKY11* in *A*. *thaliana* significantly increased tolerance to salt stress in transgenic plants. Further investigations indicated that the transgenic plants showed a lower MDA content and higher enzyme activity of POD, SOD, and CAT than those of WT under salt stress. In addition, the transgenic plants had higher contents of proline and soluble sugar compared with WT under salt stress. The above results indicate that PcWRKY11 may play a positive role in plant adaptation to salinity conditions by reducing ROS levels and increasing osmosis substance synthesis.

## 4. Materials and Methods

### 4.1. Plant Material and Treatments

*P. cuspidatum* seeds were obtained from the Beijing Botanical Garden at the Institute of Botany, Chinese Academy of Sciences, Beijing, China. *A. thaliana* Columbia ecotype (Col-0) was used to generate transgenic plants. Seeds were surface-sterilized with 75% (*v*/*v*) ethanol for 1 min and then soaked in 10% (*v*/*v*) hydrogen peroxide or 10% (*v*/*v*) sodium hypochlorite solution for 10–20 min. The seeds were washed 3 times with sterile water and sown on Murashige and Skoog (MS) medium containing 3% (*w*/*v*) sucrose and 0.8% (*w*/*v*) agar (pH 5.8). Plants were grown in a greenhouse at 22 ± 2 °C with a 16-h light/8-h dark photoperiod and 50% relative humidity. Tobacco seeds were sown in pots containing a 1:1 (*v*/*v*) mixture of nutrient soil and vermiculite. Approximately 1-month-old tobacco plants were used in the experiments [29].

The *P. cuspidatum* gene expression patterns were analyzed using the leaves of 1-month-old seedlings. The roots, stems, and leaves were collected separately for the subsequent RNA isolation and tissue-specific gene expression analysis. The following treatments were used: (1) Drought treatment: Tissue culture seedlings were transferred to MS medium containing 300 mM mannitol, and treated for 0, 2, 4, 8, 12, or 24 h. Samples were then placed in liquid nitrogen; (2) high-salt treatment: tissue culture seedlings were transferred to MS medium containing 300 mM NaCl; cultured for 0, 2, 4, 6, 8, 12, 24, or 48 h; and sampled and placed in liquid nitrogen; (3) UV-C treatment: seedlings were placed under an ultraviolet lamp at a distance of about 1 m; cultured for 0, 24, 48, 72, or 96 h; and sampled and placed in liquid nitrogen; (4) MeJA treatment: The leaves of *P. cuspidatum* sterile seedlings were sprayed with 0.1 mM MeJA; cultured for 0, 2, 4, 8, 16, or 32 h; and sampled and placed in liquid nitrogen; (5) SA treatment: the leaves of aseptic seedlings of *P. cuspidatum* were sprayed with 1 mM SA; cultured for 0, 4, 8, 12, or 24 h; and sampled and placed in liquid nitrogen; control treatment: Untreated aseptic seedlings of *P. cuspidatum* were used as a control before treatment. All treatments were completed with three replicates. The collected samples were immediately frozen in liquid nitrogen and stored at −80 °C.

### 4.2. Gene Cloning and Sequence Analysis

The *PcWRKY11* sequence was obtained by transcriptome analysis of *P. cuspidatum* under the MeJA treatment (NCBI-SRA database accession number: PRJNA626400). Total RNA was extracted from *P. cuspidatum* using the Plant Total RNA Purification Kit (GeneMark, Taiwan, China). Purified RNA (1 µg) was used as the template for synthesizing first-strand cDNA with the Hifair^®^ II 1st Strand cDNA Synthesis Kit (gDNA digester plus) (Yeasen, Shanghai, China). The PcWRKY11 open reading frame was amplified using *PcWRKY11*-F/R primers. The PCR program was as follows: 95 °C for 3 min; 35 cycles of 95 °C for 10 s, 58 °C for 30 s, and 72 °C for 30 s; 72 °C for 10 min. The PCR products were purified and cloned into the *pEASY-Blunt* cloning vector (TransGen, Beijing, China) and subjected to sequencing. Primers were designed using Primer 6.0 software. All of the primers used are listed in Supplementary Table S1. PcWRKY11 sequence was aligned with its homologs using the ClustalX2 software. The neighbor-joining method (1000 bootstrap replicates) was used to construct a phylogenetic tree with the MEGA X software [45].

The amino acid sequences of the WRKY members from other species were obtained from the EMBI database (https://www.ebi.ac.uk/ebisearch/search.ebi, accessed on 18 February 2022). i-TOL (https://itol.embl.de, accessed on 18 February 2022) and WebLogo 3 (http://weblogo.threeplusone.com, accessed on 18 February 2022) were used to visualize the homologous gene alignment results [46]. STRING (https://cn.string-db.org/cgi/network, accessed on 18 February 2022) was used to identify the protein association networks [47]. WoLF PSORT (https://wolfpsort.hgc.jp, accessed on 18 February 2022) and SUBA4 (https://suba.live, accessed on 18 February 2022) were used to predict the subcellular localization of the PcWRKY11 protein.

### 4.3. Vector Construction and Agrobacterium-Mediated A. thaliana Transformation

To meet the different experimental needs, we designed and constructed a series of vectors. The *pBI121-PcWRKY11-eGFP* fusion expression vector was used to verify the subcellular localization of PcWRKY11 in plant cells; the *pB42AD-PcWRKY11*, *pLacZi-W-box*, *pLacZi-mW-box* yeast expression vector was used for the yeast one-hybrid experiments; the *pBI121-PcWRKY11* plants expression vector was used for transformation in *A. thaliana*. The *pBI121* vector was digested with *Bam*HI and *Sac*I to construct the *pBI121-TF* expression vector; the pB42AD vector was digested with *Eco*RI and *Xho*I to construct the *pB42AD-TF* expression vector. The *Eco*RI and *Kpn*I double digestion pLacZi vector was used to construct the *pLacZi-W-Box*/*mW-Box* expression vector.

The *PBI121-PcWRKY11* vector was transfected into *A. thaliana* ecotype Columbia-0 by *Agrobacterium tumefaciens* GV3101 transformation. *A. thaliana* was transformed using the floral dip method [48]. The seeds of transformants were seeded in 1/2MS medium (containing 50 mg/L Kan) to select the transformed lines until the T3 generation. Seeds of the T3 generation of transgenic plants (OE1, OE4, OE8, randomly selected) were sown and germinated in a nutrient soil to a vermiculite ratio of 1:1 in flowerpots (diameter 10 cm) with normal management in a growth chamber at 25 ± 1 °C under a 16-h light and 8-h dark regime in parallel with the WT seeds.

The sterilized WT and 3 transgenic *A. thaliana* seeds were spread on 1/2 MS solid medium (containing 50 mg/L Kan) for 4 days after germination and growth, and the *A. thaliana* seedlings were transferred to different concentrations of NaCl. Plants were grown vertically for 5 days on 1/2 MS medium in NaCl (150 mM, 200 mM). As a control, the growth state of *A. thaliana* seedlings was observed, and the root length and fresh weight of *A. thaliana* seedlings were measured and counted. The sterilized WT and 3 transgenic *A. thaliana* seeds were spread on 1/2 MS (containing 50 mg/L Kan) medium, vernalized at 4 °C for 2–3 days, and then placed in an incubator nourish. The 13-day-old *A. thaliana* seedlings were transferred to nutrient soil: vermiculite = 1:1 soil and placed in a greenhouse under the same conditions. In total, 9 small plates (36 plates in total) of 4 *A. thaliana* plants grown in soil for 4 weeks were placed in the same plate and watered every 3 days with 300 mM NaCl solution. After 15 days of treatment, 6 seedlings were randomly selected from each line as samples, recorded and weighed, and then quickly frozen in liquid nitrogen and stored in a −80 °C refrigerator for measurement of the physiological and biochemical indicators [49].

### 4.4. Subcellular Localization Analysis of PcWRKY11

To analyze the subcellular localization, the PcWRKY11 coding sequence without the termination codon and the eGFP gene sequence were amplified, respectively, by overlapping PCR. The constructed *pBI121-PcWRKY11-eGFP* plant expression vector and the positive control vector (*pBI121-eGFP*) were inserted into separate *Agrobacterium tumefaciens* strain GV3101 cells using the freeze-thaw method. The *A. tumefaciens* cells transformed with the plant expression vectors were injected into tobacco leaves for the transient expression experiments. At 48 h after the injection, the eGFP fluorescence was observed using an LSM 880 confocal laser scanning microscope (Zeiss, Germany) and the nuclei were stained with DAPI.

### 4.5. Yeast One-Hybrid Assay

For a yeast one-hybrid assay, the full-length *PcWRKY11* coding sequence was amplified by PCR using gene-specific primers and inserted into the yeast expression vector *pB42AD* between the *Eco*RI and *Xho*I sites. Triple tandem repeats of the W-Box and mutant W-Box (mW-Box) sequences were inserted between the *Eco*RI and *Kpn*I sites of the pLacZi vector. Yeast strain EGY48 cells were co-transformed with the *pB42AD-PcWRKY11* and pLacZi-W-Box or pLacZi-mW-Box recombinant plasmids. The transformants were selected and grown on a synthetic dropout medium lacking tryptophan and uracil (SD/−Trp/−Ura). The positive yeast strains were cultured on SD/−Trp/−Ura medium containing 20 mg/mL 5-bromo-4-chloro-3-indolyl-β-D-galactopyranoside (X-gal) to analyze the color development of the strains.

### 4.6. Quantitative Real-Time PCR Analysis

For a quantitative real-time PCR (qRT-PCR) assay, 1 μL of cDNA was added to a solution consisting of Hieff® qPCR SYBR Green Master Mix (Yeasen), forward primer (1 μM), and reverse primer (1 μM), with a final volume of 10 μL. The qRT-PCR was performed on the Applied Biosystems^®^ QuantStudio™ 7 Flex Real-Time PCR System (Applied Biosystems, Waltham, MA, USA), with the following program: 95 °C for 5 min; 40 cycles of 95 °C for 10 s, 60 °C for 20 s, and 72 °C for 20 s. Relative expression levels were calculated using the 2^−ΔΔCt^ method, with *PcNDUFA13* serving as an internal reference control [50]. Details regarding the qRT-PCR primers are listed in Table S1. All qRT-PCR analyses were completed with three independent biological replicates.

### 4.7. Determination of Related Physiological Indexes

The chlorophyll content was measured according to the method of [51]. The soluble sugar content was measured according to the method of [52]. The proline content was measured according to the method of [53]. The GSH content was measured according to the method of [54]. The GSSH content was measured according to the method of [55]. POD activity was measured using the protocols described in [56]. SOD activity was measured as in [57]. CAT activity was measured following [56]. The MDA content was measured using the protocols described in [58].

### 4.8. Statistical Analysis

All data are presented herein as the mean ± standard error from three independent replicates. The significance of the differences between the mean values for the control and treated samples was assessed by 1-way analysis of variance (ANOVA) (*p* < 0.05). Statistical differences were considered significant when * *p* ≤ 0.05, ** *p* ≤ 0.01. The GraphPad Prism 8 program was used for the data analyses. Figures were produced with the Microsoft Office 365 and Adobe Photoshop CS5 program.

## Data Availability

Not applicable.

## Supplementary Materials

The following are available online at https://www.mdpi.com/article/10.3390/ijms23084357/s1.
