# Supplementary material for: PcWRKY11, an II-d WRKY Transcription Factor from Polygonum cuspidatum, Enhances Salt Tolerance in Transgenic Arabidopsis thaliana
Source: Int J Mol Sci. 2022 Apr 14;23(8):4357. doi: 10.3390/ijms23084357 (PMC9025145; doi:10.3390/ijms23084357)
Supplement: Supplementary file 1 [file ijms-23-04357-s001.zip › ijms-1668531-supplementary.pdf]

International Journal of
Molecular Sciences

MDPI

Figure S1

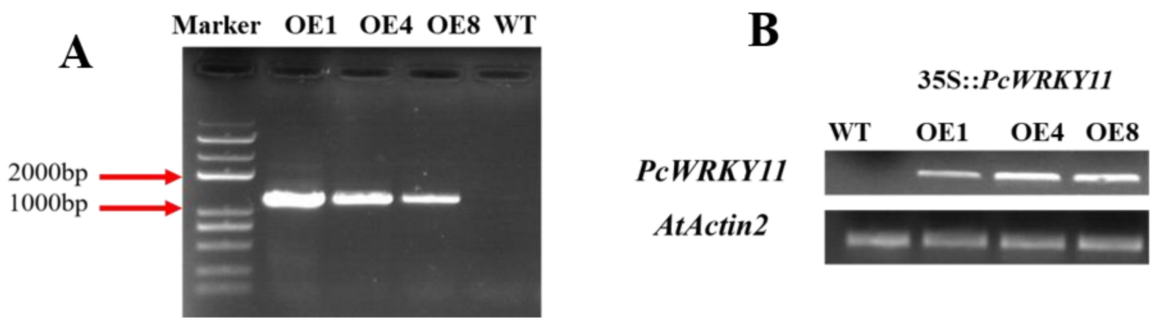

Figure S1. Identification of transgenic *A. thaliana* on DNA and RNA level by PCR. (A) PCR identification on the DNA level of candidate lines. (B) Identification of transgenic plants on RNA level by semi-quantitative RT-PCR. The reference gene of RT-PCR is Atactin2; WT, wild type plants; OE1, OE4, OE8, the selected lines for the following expriments.

Figure S2

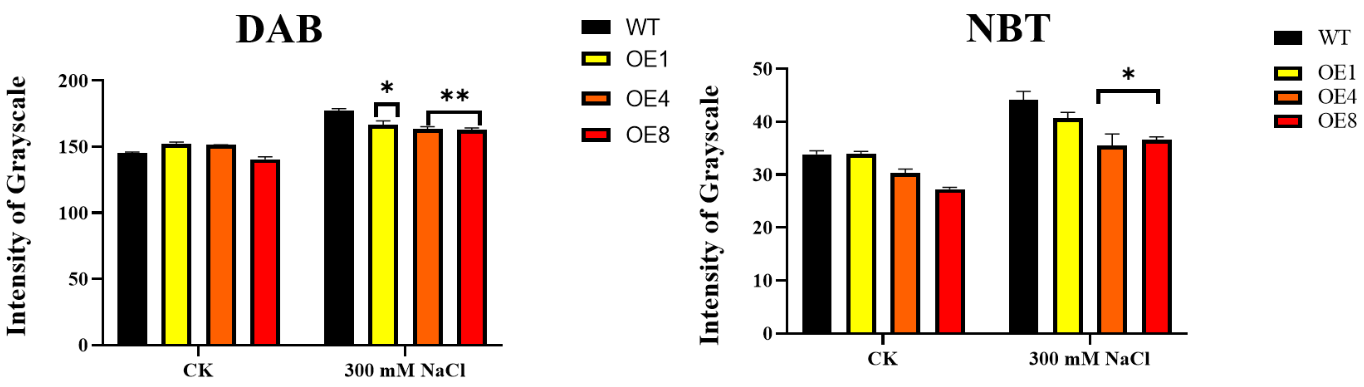

Figure S2. Quantitative analysis of staining results in Figure 5A.
The mean values and standard errors were calculated with three independent experiments. One, two and three asterisks represent significance level P<0.05, P<0.01 and P<0.001; WT, wild type plants; OE, over-expressed plants; CK, under normal conditions.

Figure S3

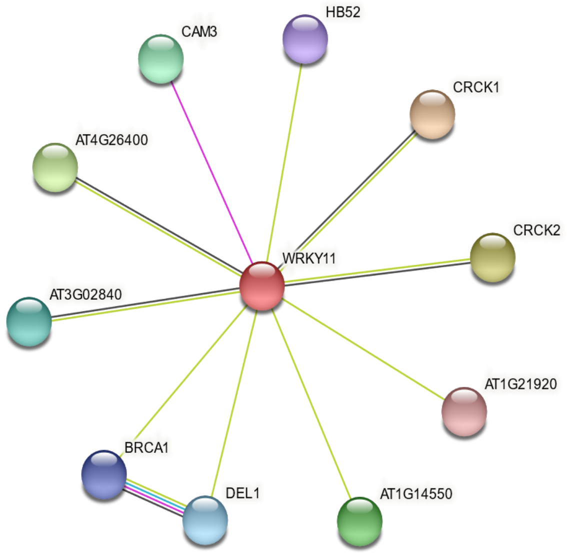

Figure S3. AtWRKY11 protein STRING interaction network.

Table S1. Primer sequences used in this study.

Primer	Sequence (5'-3')
PcWRKY11-F	ATGGAGATCCAGCTCCCCG
PcWRKY11-R	TTAATTTAATGTCCCCAACGCTGC
PcWRKY11-QPCR-F	AGTCTTTCAGCCTGTCGTCG
PcWRKY11-QPCR-R	GGCTTCTGGCCGTACTTTCT
PcNDUFA13-F	ATGTACCAGGTCGGCGTAGG
PcNDUFA13-R	TCCTTCATAATTCTGGCTTCC
pBI121-PcWRKY11-F	CGGGGGACTCTAGAGGATCCAATGGAGATCCAGCTCCCCG
pBI121-PcWRKY11-R	GATCGGGGAAATTCGAGCTCTTACTTGTACAGCTCGTCCA
AtActin2-F	GGTAACATTGTGCTCAGTGGT
AtActin2-R	GCATCAATTCGATCACTCAGAG

AtP5CS1-F	AGCTTGATGACGTTATCGATCT
AtP5CS1-R	AGATTCCATCAGCATGACCTAG
AtSOD-F	AGGAAACATCACTGTTGGAGAT
AtSOD-R	GAGTTTGGTCCAGTAAGAGGAA
AtAPX-F	GATGTCTTTGCTAAGCAGATGG
AtAPX-R	GAGTTGTCGAAGATTAGAGGGT
PcWRKY11-EGFP-	ACACGGGGGACTCTAGAGGATCC
pBI121-F	ATGGAGATCCAGCTCCCCG
PcWRKY11-EGFP-	GAACGATCGGGGAAATTCGAGCTC
pBI121-R	TTAATTTAATGTCCCCAACGCTGC
PcWRKY11-pB42AD-F	GATTATGCCTCTCCCGAATTCATGGAGATCCAGCTCCCCG
PcWRKY11-pB42AD-R	CTTCTCGAGTCGGCCGAATTCTTAATTTAATGTCCCCAACGCTGC
W-box-F	AATTCTTGACCCCTTTTGACCCGTTTTGACCTCCAGGTAC
W-box-R	CTGGAGGTCAAAACGGGTCAAAAGGGGTCAAG
mW-box-F	AATTCTAGACCCCTTTAGACCCGTTTAGACCTCCAGGTAC
mW-box-R	CTGGAGGTCTAAACGGGTCTAAAGGGGTCTAG
